# Significantly Improved Dielectric Performance of Poly(1-butene)-Based Composite Films via Filling Polydopamine Modified Ba(Zr_0.2_Ti_0.8_)O_3_-Coated Multiwalled Carbon Nanotubes Nanoparticles

**DOI:** 10.3390/polym13020285

**Published:** 2021-01-17

**Authors:** Lingfei Li, Qiu Sun, Xiangqun Chen, Zhaohua Jiang, Yongjun Xu

**Affiliations:** 1 School of Chemistry and Chemical Engineering, Harbin Institute of Technology, Harbin 150001, China; lilingfei3473@126.com (L.L.); xuyongjun1218@126.com (Y.X.); 2 School of Materials Science and Engineering, Harbin Institute of Technology, Harbin 150001, China; chenxq@hit.edu.cn

**Keywords:** sol–gel hydrothermal method, PDA-modified BZT@MWCNTs nanoparticles, dielectric performance, synergistic effect

## Abstract

The low dielectric constant of the nonpolar polymer poly(1-butene) (PB-1) limits its application as a diaphragm element in energy storage capacitors. In this work, Ba(Zr_0.2_Ti_0.8_)O_3_-coated multiwalled carbon nanotubes (BZT@MWCNTs) were first prepared by using the sol–gel hydrothermal method and then modified with polydopamine (PDA) via noncovalent polymerization. Finally, PB-1 matrix composite films filled with PDA-modified BZT@MWCNTs nanoparticles were fabricated through a solution-casting method. Results indicated that the PDA-modified BZT@MWCNTs had good dispersion and binding force in the PB-1 matrix. These characteristics improved the dielectric and energy storage performances of the films. Specifically, the PDA-modified 10 vol% BZT@ 0.5 vol% MWCNTs/PB-1 composite film exhibited the best dielectric performance. At 1 kHz, the dielectric constant of this film was 25.43, which was 12.7 times that of pure PB-1 films. Moreover, its dielectric loss was 0.0077. Furthermore, under the weak electric field of 210 MV·m^−1^, the highest energy density of the PDA-modified 10 vol% BZT@ 0.5 vol% MWCNTs/PB-1 composite film was 4.57 J·cm^−3^, which was over 3.5 times that of PB-1 film (≈1.3 J·cm^−3^ at 388 MV·m^−1^).

## 1. Introduction

Poly(1-butene) (PB-1), which exhibits low dielectric loss (0.0005 at 1 kHz) [1], high mechanical properties, chemical stability, and creep resistance [2], has a good potential to be used as a diaphragm for storage capacitors [3]. However, PB-1, a nonpolar polymer, has a very low dielectric constant of no more than 2.1 at 100–30 MHz [4].

The dielectric properties of PB-1 are usually improved through the formation of crystal type I by adding functional nanometer fillers, such as ceramic particles or metal or carbon-based materials [5,6,7]. These fillers have their own disadvantages. For example, ceramic particles, such as BaTiO_3_ (BT), Ba_0.2_Sr_0.8_TiO_3_, and CaCu_3_Ti_4_O_12_ [8,9,10,11], must be added at high amounts to improve the dielectric properties of polymer matrixes. This requirement greatly reduces mechanical properties. Although adding a low content of conductive metal [12] or carbon-based materials (carbon black, multiwalled carbon nanotubes (MWCNTs), and graphene nanosheets) [13,14,15] can considerably increase the dielectric constant around the permeability threshold, this approach induces leakage currents and reduces breakdown strength. Adding a small amount of conductors, which can increase the dielectric constant of composite polymers while keeping the dielectric loss low, is an effective way to reduce the amount of ceramic fillers in polymer composites [16,17,18,19,20]. Dang et al. [21,22] found that for poly(vinylidene fluoride) (PVDF) matrix composites, the dielectric constant increases and the dielectric loss reduces due to the synergistic effect of BT particles and conductive MWCNTs. They then proposed the dielectric mechanism model. Kim, H. et al. [23] prepared BT/CNT/PVDF composites through melt deposition modeling and 3D printing. The most desirable combination of the dielectric constant and dielectric loss (118 and 0.11 at 1 kHz) was exhibited by composites containing 1.7 wt% CNT/45 wt% BT/PVDF. However, breakdown field strength drastically decreased, and energy storage density was insufficient.

In addition, when ceramics and polymers are simply mixed together, the conductor phase agglomerates, and interface adhesion between inorganic nanoparticles and the polymer matrix is weak due to their poor compatibility, which degrades the dielectric properties and mechanical properties of the materials [24]. Thus, modifying the surfaces of nanopacking with hydrogen peroxide [25], silane coupling agents [26], polyvinylpyrrolidone [27], or polydopamine (PDA) [28] has become a research hotspot. Among these materials, PDA performs well in the surface modification of ceramic particles. It can improve dispersion and compatibility between fillers and the polymer matrix, thus enhancing the interaction between interfaces and reducing the hole defects of interfaces. PDA can form an insulating interlayer between nanofillers and the polymer matrix, thus restricting the movement of charge carriers at the interface or in the polymer matrix and then reducing the dielectric loss of the composite film [29,30,31].

Barium zirconate titanate (BZT) is an ABO_3_ perovskite-type dielectric nonlinear material with low leakage current and high pressure resistance [32]. In this work, we investigated the preparation and dielectric performance of the PB-1 matrix composite films filled with PDA-modified BZT-coated MWCNTs (BZT@MWCNTs). BZT@MWCNTs were prepared via a sol–gel hydrothermal method and then modified with PDA to improve their interface interaction and compatibility with the PB-1 matrix. The PB-1 matrix composite films filled with the PDA-modified BZT@MWCNTs nanoparticles were fabricated through a solution-casting method. The composition and morphologies of the samples were characterized by utilizing X-ray diffraction (XRD) analysis, scanning electron microscopy (SEM), X-ray photoelectron spectroscopy (XPS), and transmission electron microscopy (TEM). The effects of the PDA-modified BZT@MWCNTs on the dielectric properties of PB-1 composite films were investigated.

## 2. Materials and Methods

### 2.1. Materials

PB-1 (type PB0110M) was purchased from Lyondellbasell (Hoofddorp, the Netherlands). MWCNTs (20–40 nm in diameter, purity: N97%) were purchased from Shenzhen Nanotech Port Co., Ltd. (Shenzhen, China). Decalin, dopamine hydrochloride, tris(hydroxymethyl)aminomethane, acetic acid, barium, acetic acid, tetrabutyl titanate, ethylene glycol methyl ether, and sodium hydroxide were purchased from Aladdin Reagent Co., Ltd. (Shanghai, China). Zirconium isopropyl alcohol was purchased from Alfa Aesar Co., Ltd. (Tianjin, China).

### 2.2. Preparation of BZT@MWCNTs Nanoparticles

**Figure 1:**
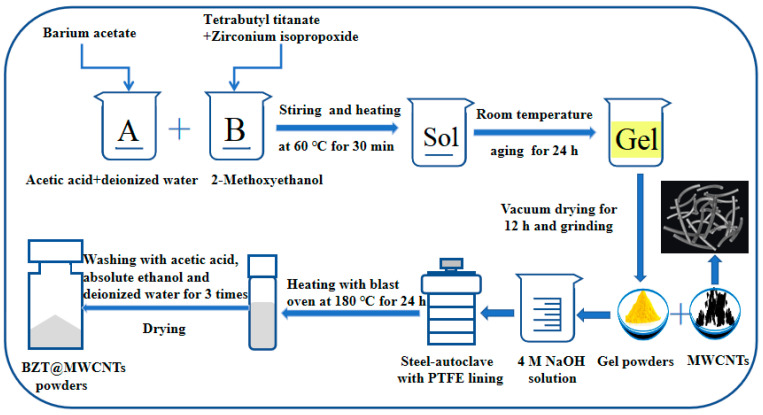
Preparation of Ba(Zr_0.2_Ti_0.8_)O_3_-coated multiwalled carbon nanotubes (BZT@MWCNTs) nanoparticles.

First, barium acetate was dissolved in acetic acid and deionized water to obtain the A solution. Tetrabutyl titanate was mixed at a certain stoichiometric ratio with isopropanol zirconium, and then glycol methyl ether was added to the mixture to obtain the B solution. The A and B solutions were mixed to obtain the initial sol. Then, a light yellow gel was formed via sol–gel transition. After the continuous evaporation of ethylene glycol methyl ether and acetic acid at 120 °C for 12 h, the dried gel was obtained and then mortared into a powder as a precursor for the following hydrothermal synthesis. The precursor was proportioned with carbon nanotubes and transferred to a 50 mL stainless steel autoclave lined with Teflon and filled with an aqueous solution of 4 mol·L^−1^ NaOH. The autoclaves were heated at 180 °C under autogenous pressure for 24 h and then naturally cooled to room temperature. The solid precipitate was centrifuged; washed successively with acetic acid, ethanol, and distilled water; and then dried at 100 °C for 12 h. BZT@MWCNTs nanoparticles were thus obtained. The preparation procedure is shown in Figure 1.

### 2.3. Surface Modification of BZT@MWCNTs Nanoparticles

Aqueous solutions of dopamine hydrochloride and Tris reagent were prepared. Then, BZT@MWCNTs were added into the aqueous solutions, and the pH of the solutions were adjusted to 8.5. Then, the solutions were ultrasonically treated in an ice water bath for 30 min and stirred at room temperature for 24 h [33]. Finally, the PDA-coated BZT@MWCNTs were repeatedly washed until colorless via centrifugal separation in deionized water, and then dried to a constant weight in a vacuum oven at 60 °C. The modified samples were labeled as PDA-modified BZT@MWCNTs.

### 2.4. Preparation of PDA-Modified BZT@MWCNTs/PB-1 Composite Films

PB-1 resin was dissolved quickly in decalin solvent at 130 °C to produce an 8% (by mass percentage) solution of PB-1 [7]. The PDA-modified BZT@MWCNTs nanoparticles were dispersed in decalin solvent via ultrasonication for 1 h. After mixing the colloidal dispersion of PDA-modified BZT@MWCNTs and PB-1 polymer solution for 3 h under full agitation, ultrasonic defoamination was performed for 1 h. The defoamed solution was poured onto a glass plate and extended to obtain a uniform initial film. The PDA-modified BZT@MWCNTs/PB-1 composite film was obtained by annealing at 80 °C for 24 h and heating to 100 °C for 24 h under vacuum. BZT/PB-1 and BZT@MWCNTs/PB-1 composite films were prepared for comparison via the same method above.

### 2.5. Characterization

XRD measurements were performed by using a Phillips X’Pert Pro X-ray diffractometer (PANalytical B.V., Almelo, The Netherlands) with Cu Kα radiation in the 2θ range, from 10° to 70° at a rate of 5°·min^−1^ (30 kV, 20 mA, λ = 0.15406 nm). The morphologies of the samples were investigated with a scanning electron microscope at 20 kV (S-4800 model, Japan Hitachi, Tokyo, Japan) after being sprayed with gold by using the Gatan Precision Etching Coating System (Model 682, Gatan, Pleasanton, CA, USA). The elemental valence analysis of PDA-modified samples was conducted via XPS with an ESCALAB250XI model X-ray photoelectron spectroscope (Thermo Fisher, Waltham, MA, USA) with Al Kα radiation. The survey spectra of the samples were collected over the range of 0−1000 eV. TEM observation was performed by using a JEM-2100 instrument (Japan Hitachi, Tokyo, Japan) with an accelerating voltage of 200 kV. The dielectric properties and conductivity of samples were analyzed at room temperature by using a broadband dielectric spectrometer (model Novocontrol GmbH Concept 40, Karlsruhe, Germany) over the frequency range of 10^2^ Hz to 10^6^ Hz. Breakdown strength (E) was tested with a voltage tester (TH9102B, Tonghui Electronics, Changzhou, China) under DC 1000 V·s^−1^ until the sample broke down. The electric displacement versus electric field (P–E) loops of the composite films were characterized by using a Premier II ferroelectric test system (Radiant Technologies, Albuquerque, NM, USA) at 1 kHz.

## 3. Results

### 3.1. Characterization of BZT@MWCNTs Nanoparticles

**Figure 2:**
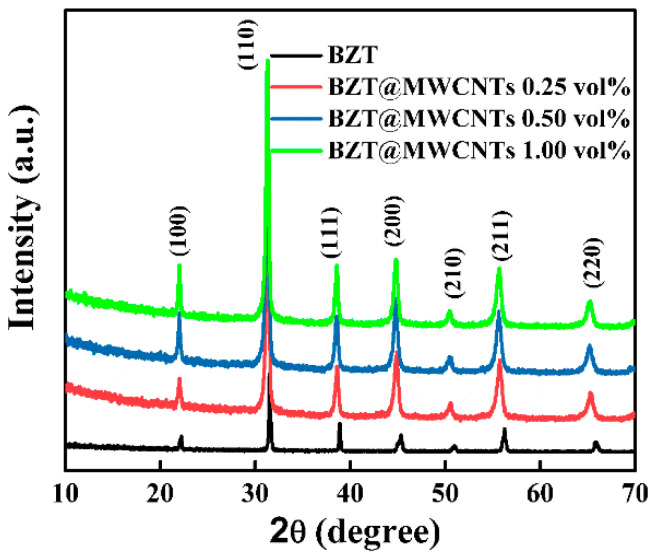
XRD patterns of BZT@MWCNTs nanoparticles with different MWCNTs contents.

BZT was prepared through the traditional sol–gel method, followed by high-temperature calcination [34,35]. Pure BZT@MWCNTs were difficult to obtain, given that calcination must be performed under anaerobic conditions as the MWCNTs were added. In this experiment, BZT@MWCNTs nanoparticles with different MWCNTs contents were prepared via a sol–gel hydrothermal method, with water heat treatment instead of high-temperature calcination. The XRD results in Figure 2 show the characteristic diffraction peaks of BZT crystals at (100), (110), (111), (200), and (211), indicating that the powder was the pure phase of the BZT perovskite without other heterocrystals [36]. After the addition of MWCNTs, no new diffraction peak was observed, and the intensity of the characteristic diffraction peaks of BZT increased with the addition of MWCNTs, indicating that the addition of MWCNTs promoted the crystallization of BZT.

**Figure 3:**
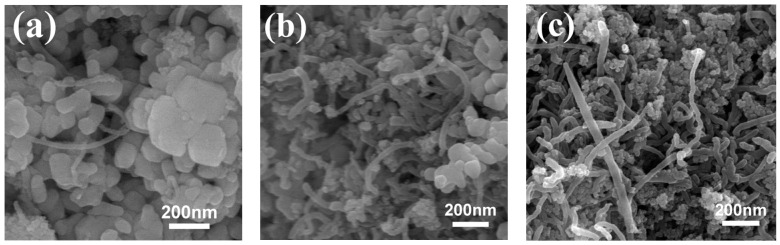
SEM images of BZT@MWCNTs nanoparticles with different MWCNTs contents: (**a**) 0.25 vol%, (**b**) 0.50 vol%, and (**c**) 1.00 vol%.

The morphology of BZT@MWCNTs nanoparticles with different MWCNTs contents are shown in Figure 3. The BZT grains were relatively uniform, and the BZT crystals decreased in size and became evenly dispersed around MWCNTs as the MWCNTs content increased. When the amount of MWCNTs was 1.00 vol%, the growth rate of the BZT grains decelerated, inducing the formation of small BZT particles in a nonthermodynamic stable state that facilitated agglomeration [37].

### 3.2. Characterization of PDA-Modified BZT@MWCNTs Nanoparticles

**Figure 4:**
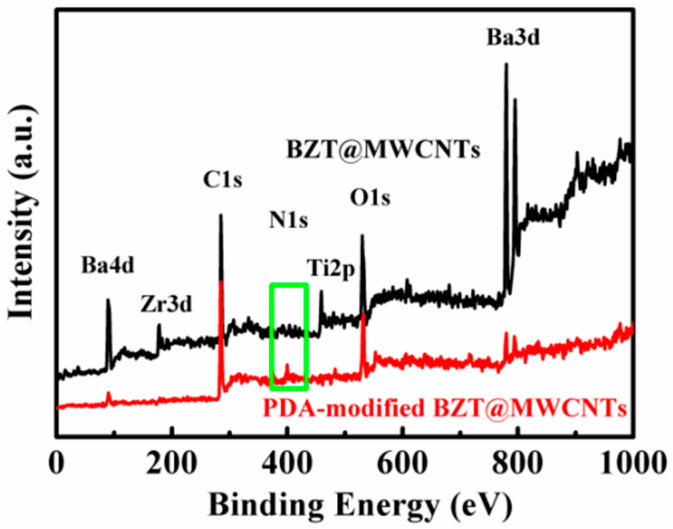
XPS spectra of BZT@MWCNTs and PDA-modified BZT@MWCNTs nanoparticles.

The surfaces of BZT@MWCNTs nanoparticles were modified with PDA to improve the compatibility between BZT@MWCNTs nanoparticles and the PB-1 polymer matrix. Figure 4 shows the XPS results of BZT@MWCNTs and PDA-modified BZT@MWCNTs nanoparticles when MWCNTs were added at the amount of 0.5 vol%. The sample before modification almost had no N 1s peaks. After PDA treatment, the N 1s peak corresponding to free -NH_2_ appeared at 399.0 eV. The surface coating shielding of PDA greatly reduced the signals of Ba 3d, Ba 4d, Zr 3s, Ti 2p, and C 1s electrons from BZT@MWCNTs. This result suggested that PDA had been coated on the surfaces of BZT@MWCNTs.

**Figure 5:**
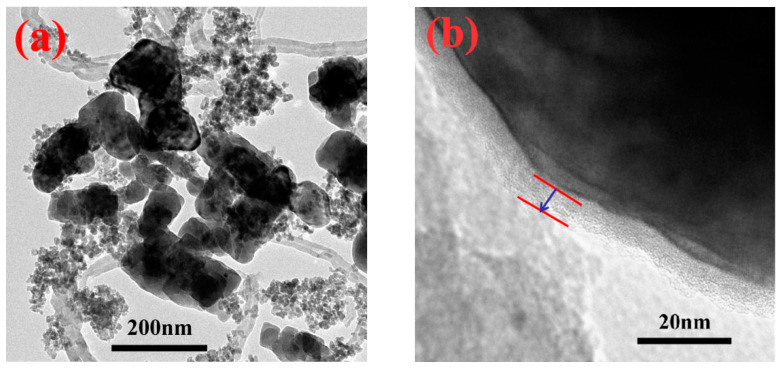
(**a**,**b**) TEM images with different magnifications of PDA-modified BZT@MWCNTs nanoparticles. The arrow and horizontal lines mark the thickness of the PDA layer.

As shown in Figure 5, TEM characterization was performed to study the effect of PDA on the dispersion of BZT@MWCNTs. As could be seen from Figure 5a, BZT@MWCNTs were dispersed evenly after modification with PDA, and BZT grains were uniformly dispersed on the surfaces or surroundings of MWCNTs. As illustrated in Figure 5b, PDA was evenly coated on the surfaces of BZT@MWCNTs with an average thickness of 8 nm.

### 3.3. Morphology of PDA-Modified BZT@MWCNTs/PB-1 Composite Films

**Figure 6:**
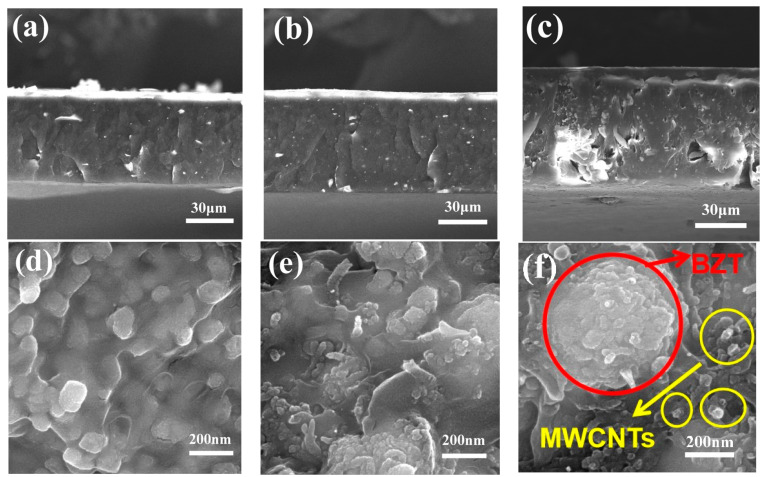
Cross-section morphology of composite films with (**a**) 0.25 vol%; (**b**) 0.50 vol%; and (**c**) 1.00 vol% MWCNTs. The scale bar is 30 μm. (**d**–**f**) are the high-resolution images of (**a**–**c**), respectively. The scale bar is 200 nm.

The morphologies of the PB-1 composite films with PDA-modified BZT@MWCNTs (10 vol% BZT and 0–1.00 vol% MWCNTs) nanoparticles were observed through SEM, as shown in Figure 6. The reason why 10 vol% was selected as the appropriate addition amount of BZT is explained in Figures S1–S3 and Table S1.

Figure 6a–c shows the fracture surface morphology of the PDA-modified BZT@MWCNTs/PB-1 composite films with different MWCNTs contents. When the added amount of MWCNTs did not exceed 0.5 vol%, the cross-section of the composite film was flat, and the film thickness was relatively uniform. The average thickness of the composite films was approximately 52 μm. When the MWCNTs content was increased to 1.00 vol%, pores appeared on the fracture surface due to the poor compatibility between the excessive fillers and the PB-1 matrix, and the average thickness of the composite film increased to 68 μm. Figure 6d–f show the partial enlargement of the cross-sections of the PDA-modified BZT@MWCNTs/PB-1 composite films with different MWCNTs contents. The MWCNTs uniformly dispersed around BZT particles when their content was less than 0.5 vol%. In the sample with 1.0 vol% MWCNTs, the amount of MWCNTs increased and BZT nanoparticles agglomerated, which could induce the deterioration of the dielectric properties of the composite film.

### 3.4. Dielectric Properties of BZT@MWCNTs/PB-1 Composite Films

**Figure 7:**
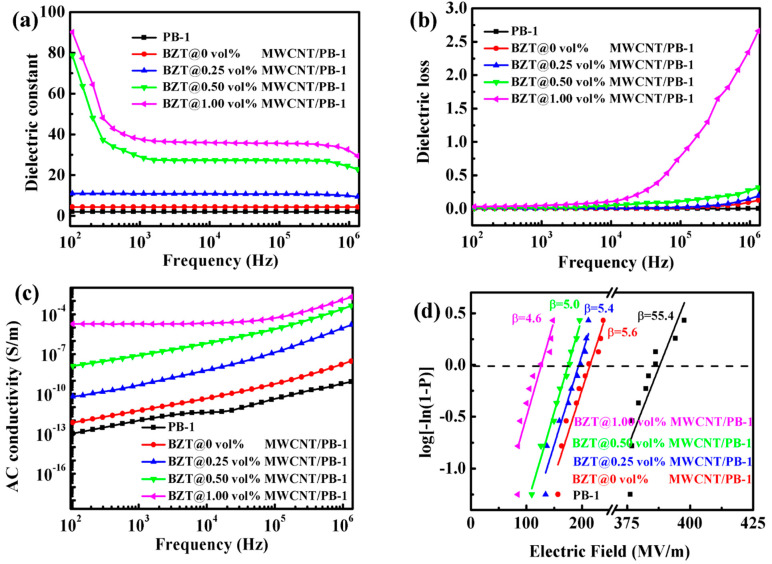
Variation in (**a**) dielectric constant, (**b**) dielectric loss, and (**c**) AC conductivity with frequency shown by the 10 vol% BZT@MWCNTs/PB-1 composite films with different MWCNTs contents at room temperature. (**d**) Effects of the addition of different MWCNTs contents to 10 vol% BZT on the breakdown performance of the composite films.

The frequency-dependent behaviors of the dielectric constant (ε’) and dielectric loss (tan δ) of the 10 vol% BZT@MWCNTs/PB-1 composite films with various volume fractions of MWCNTs were investigated as shown in Figure 7. The 10 vol% BZT/PB-1 and PB-1 films were used for comparison.

As shown in Figure 7a, when the content of MWCNTs, the third component of the BZT@MWCNTs/PB-1 composite film, was low, the ε’ of the composite film showed reduced dependency on frequencies from 10^2^ to 10^6^ Hz. The trend of this film was similar to that of pure PB-1 film. The ε’ of the composite film with 0.5 vol% MWCNTs decreased at low frequencies and then stabilized from 10^3^ to 10^5^ Hz. It dropped slightly at 10^6^ Hz likely because the rotation of the dipole was slower than the change in the electric field. Thus, the contribution of relaxation polarization, such as interfacial polarization, to ε’ reduced slightly with the increase in frequency. At 1 kHz, the ε’ of the composite film with 0.5 vol% MWCNTs was 28.40, which was 6.5 times that of the sample without MWCNTs and 14.1 times that of the pure PB-1 film. The ε’ of the composite film tended to increase with the increase in the amount of MWCNTs. Frequency-dependent behavior is related to interface polarization [38]. Although BZT and BZT@MWCNTs could enhance interface polarization, the BZT@MWCNTs ternary composite film exhibited strong frequency dependence. In addition, the ε’ of this film was considerably higher than that of the binary composite film with only BZT, because the addition of MWCNTs enhanced the accumulation and migration of charges at the interface [39]. In addition, a large number of microcapacitor structures were formed inside the PB-1 matrix, greatly increasing the ε’ of the ternary composite film with MWCNTs.

As shown in Figure 7b, the tan δ of the composite films with no more than 0.5 vol% MWCNTs remained under 0.35 over the whole tested frequency range. However, as the added amount of MWCNTs increased to 1.0 vol%, tan δ changed significantly with the increase in frequency, especially at the high frequencies of 10^4^–10^6^ Hz, likely because pores and defects in the composite film increased with the increase in MWCNTs content. These structures gave rise to ionization and interface losses and subsequently increased tan δ. As shown in Figure 7c, the AC conductivity of the composite films increased with the increase in frequency and MWCNTs contents. The sample with 1.0 vol% MWCNTs had the highest electrical conductivity, i.e., 1.86 × 10^−5^ at 1 kHz, suggesting that a conductive network had formed in the composite film. This phenomenon prompted the transformation of the composite from an insulator to a semiconductor.

Breakdown strength is also an important index for measuring the energy storage density of composite films. The effects of the BZT@MWCNTs nanoparticles on the breakdown strength of the composite films are shown in Figure 7d. The reliability of the breakdown characteristics of the BZT@MWCNTs/PB-1 composite films were evaluated by using the two-parameter Weibull distribution with the following formula [40]:

(1) $$P=1-\exp[-\left({E/E}_{b}{)}^{\beta }\right],$$

where P represents the cumulative probability of electric failure; E represents the tested experimental breakdown strength; Eb represents the breakdown strength of the PB-1 or BZT@MWCNTs/PB-1 composite films at a cumulative breakdown probability of 63.2%; and β represents the shape parameter, which is related to the reliability of the films. The tested β values of all samples were greater than 1, indicating that the test results were reliable. When the added amount of MWCNTs was 1.0 vol%, the breakdown field strength of the composite films decreased with the increase in the MWCNTs volume fraction of BZT@MWCNTs nanoparticles and drastically reduced to 126 MV·m^−1^, which was considerably lower than that of PB-1 (388 MV·m^−1^). This change might be attributed to the following reasons: although adding BZT@MWCNTs fillers could give rise to defects in the polymer matrix, the MWCNTs fillers had good dispersibility in the PB-1 polymer matrix when added at low contents. Moreover, the presence of BZT hindered contact between MWCNTs, and MWCNTs also prevented the agglomeration of BZT, resulting in the negligible decrement in breakdown field strength. When MWCNTs were added at the amount of 1.0 vol%, the distance between MWCNTs, which could form a local conductive network in the PB-1 matrix, was shortened. In addition, the shape factor β, which is used to describe the uniformity and stability of films, was greatly reduced, leading to a sharp drop in breakdown strength. Thus, the PB-1 composite film with 10 vol% BZT@0.5 vol% MWCNTs had the best dielectric performance. Specifically, its ε’ and tan δ at 1 kHz were 28.40 and 0.014, respectively.

### 3.5. Dielectric Properties of PDA-Modified BZT@MWCNTs/PB-1 Composite Films

**Figure 8:**
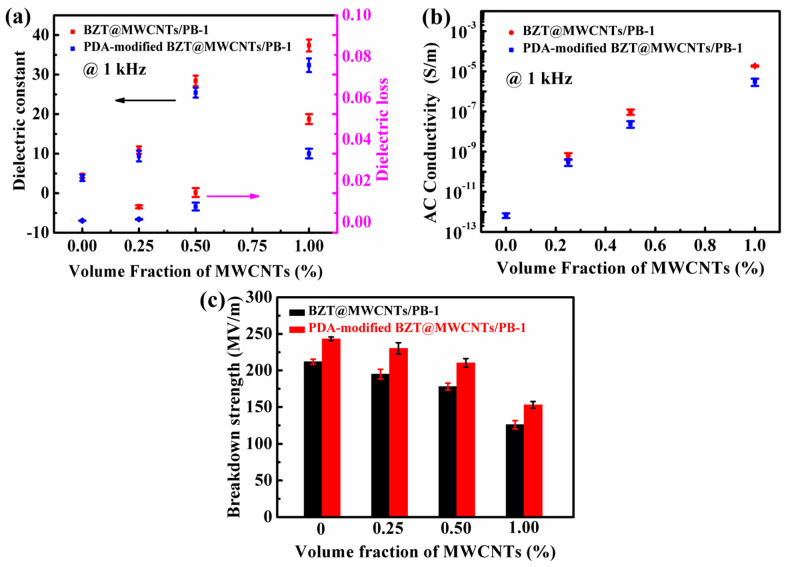
Effects of PDA modification on (**a**) the dielectric constant and dielectric loss, (**b**) AC conductivity, and (**c**) breakdown characteristics of the BZT@MWCNTs/PB-1 composite films with different MWCNTs contents.

As shown in Figure 6, PDA surface modification improved the dispersion of BZT@MWCNTs in the PB-1 matrix, increased interactions between interfaces, and reduced pore defects. These effects could influence the dielectric properties of BZT@MWCNTs/PB-1 (10 vol% BZT) composite films. Figure 8a illustrates the effects of PDA modification on the ε’ and tan δ of BZT@MWCNTs/PB-1 composite films with different MWCNTs contents at 1 kHz. ε’ and tan δ continuously increased with the increase in MWCNTs contents. The ε’ of the sample with PDA modification and 0.5 vol% MWCNTs was 25.43, which was approximately 12.7 times higher than that of pure PB-1 film and slightly lower than that of the sample without PDA modification. These differences could be attributed to reduced interfacial polarization. At the same time, the tan δ of the sample with PDA modification was 0.0077, which was considerably lower than that of the sample without PDA modification because the PDA layer enhanced the insulation of the BZT@MWCNTs nanoparticles, thus confining the accumulation and mobility of filler-matrix space charges. The effects of PDA modification on the AC conductivity at 1 kHz of the BZT@MWCNTs/PB-1 composite films with different volume fractions of MWCNTs are shown in Figure 8b Among the samples with the same loading content, the PDA-modified sample showed lower AC conductivity than the sample without PDA modification, because the PDA layer prevented the formation of conductive paths at the interface between BZT@MWCNTs and the PB-1 matrix.

Figure 8c presents the effects of PDA modification on the breakdown strength of the composite films with different volume fractions of MWCNTs. Compared with the samples without PDA modification, the PDA-modified samples had higher breakdown strength under the same conditions. Although the breakdown strength of the composite films decreased with the increase in MWCNTs content, the values of the PDA-modified samples all exceeded 150 MV·m^−1^ because of the improvement in compatibility between BZT@MWCNTs nanoparticles and the PB-1 matrix via PDA modification. In addition, the PDA layer could prevent the movement of the charge carriers in the interface between the filler and matrix, leading to reductions in tan δ and leakage current densities.

The dielectric property data of pure PB-1 film, binary composite films with BZT/PB-1 or MWCNTs/PB-1 (from our previous research work), and ternary composite films with BZT@MWCNTs/PB-1 and PDA-modified BZT@MWCNTs/PB-1 were obtained to further verify the synergistic effects of BZT, MWCNTs, and PDA on the PB-1 matrix. The data are listed in (Table 1). The experimental test frequency was 1 kHz, and the contribution of each component to ε’ was calculated in accordance with the method reported by Ning. et al. [41]. Notably, the sum of the ε’ of the binary composite films BZT/PB-1 and MWCNTs/PB-1 was far less than that of the ternary composite film under the same test conditions. In each composite film, the contribution of ε’ from the PB-1 matrix was 2.01. The contribution of BZT was calculated by using

Δ _BZT_ = ε’_BZT/PB-1_ − ε’_PB-1_, (2)

and that of MWCNTs was calculated with

Δ _MWCNTs_ = ε’_MWCNTs/PB-1_ − ε’_PB-1_. (3)

ε’_BZT/PB-1_ and ε’_MWCNTs/PB-1_ were calculated by using the dielectric constant of the 10 vol%BZT/PB-1 binary composite film shown in Figure 7a and the dielectric constant of 0.5 vol% MWCNTs/PB-1 from a reference [7], and were found to be 4.40 and 4.92, respectively. These results indicated that the contribution of BZT to the ε’ of the composite film was 2.39, and that of MWCNTs to the ε’ of the composite film was 2.91.

The contribution of BZT@MWCNTs and PDA-modified BZT@MWCNTs could be calculated by using the same equation

Δ _BZT@MWCNTs_=ε’_BZT@MWCNTs/PB-1_ − ε’_PB-1_ (4)

from Figure 7a. ε’_BZT@MWCNTs/PB-1_ was 28.40, indicating that the contribution of BZT@MWCNTs to the *ε’* of the composite film was 26.39. ε’ _PDA-modified BZT@MWCNTs_ was found to be 25.43 by using the equation

Δ _PDA-modified BZT@MWCNTs_ = ε’ _PDA-modified BZT@MWCNTs/PB-1_ − ε’_PB-1_ (5)

from Figure 8a. This result indicated that the contribution of PDA-modified BZT@MWCNTs nanoparticles to the *ε’* of the composite film was 23.42. Interestingly, Δ _BZT@MWCNTs_ > Δ _PDA-modified BZT@MWCNTs_ > Δ _BZT_ + Δ _MWCNTs_. The extent of the synergistic effect between BZT and MWCNTs was 21.09, which was calculated by using the equation

Δ _synergistic effect_ = Δ _BZT@MWCNTs_ − Δ _BZT_ − Δ _MWCNTs_. (6)

The synergy among BZT, MWCNTs, and PDA in the PDA-modified BZT@MWCNTs was 18.12, as calculated via the same calculation method above. This result illustrated that the effects of the PDA-modified BZT@MWCNTs on the dielectric properties of the composite films were very different from those of the simply blended fillers. In addition, the dielectric constant of the composite films followed the order of BZT@MWCNTs/PB-1 > PDA-modified BZT@MWCNTs/PB-1 > MWCNTs/PB-1 > BZT/PB-1, and the dielectric loss of the composite films followed the order of MWCNTs/PB-1 > BZT@MWCNTs/PB-1 > PDA-modified BZT@MWCNTs/PB-1 > BZT/PB-1, suggesting that BZT, MWCNTs, and PDA had synergistic effects on the improvement in the dielectric properties of PB-1 composite films.

**Figure 9:**
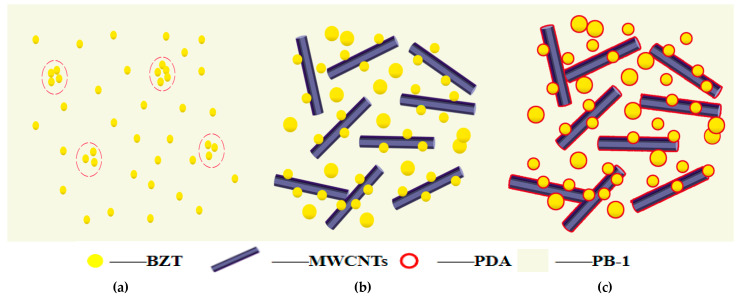
Schematic of the dispersion of fillers in the PB-1 matrix. In this schematic, (**a**–**c**) are composite films formed by adding BZT, BZT@MWCNTs, or PDA-modified BZT@MWCNTs, respectively.

The corresponding mechanism diagram was generated and is shown in Figure 9 to understand the relationship between the microstructure and dielectric properties of the PDA-modified BZT@MWCNTs/PB-1 composite film. The distribution of the filler in the matrix was a key factor for improving dielectric properties. As shown in Figure 9a, BZT nanoparticles aggregated when BZT was added alone to the PB-1 matrix at high amounts. Therefore, the third component, namely MWCNTs, was simultaneously introduced into the PB-1 matrix via BZT@MWCNTs nanoparticles that were synthesized through the sol–gel hydrothermal method. The MWCNTs could prevent the agglomeration of BZT, whereas the presence of BZT could hinder contact between MWCNTs, as depicted in Figure 9b.

As shown in Figure 9c, the surfaces of BZT@MWCNTs nanoparticles were modified with PDA to further improve the dispersion of nanoparticles in the PB-1 matrix. The improved dielectric properties of the PDA-modified BZT@MWCNTs/PB-1 composite film were attributed to the following reasons. First, in accordance with the microcapacitor model, every two adjacent MWCNTs could be regarded as local nanocapacitors. The high ε’ of the PDA-modified BZT@MWCNTs/PB-1 composite film was ascribed to the existence of a large number of microcapacitors instead of to the formation of a conductive network. BZT grains could be dispersed around or on the surfaces of MWCNTs via sol–gel hydrothermal synthesis. These grains hindered the direct contact of MWCNTs and thus prevented MWCNTs from directly connecting to each other. Second, the introduction of the PDA insulation layer could effectively improve binding capability between BZT@MWCNTs nanoparticles and the PB-1 matrix. This effect confined the accumulation and mobility of the filler–matrix space charge. Therefore, the dielectric loss of the PDA-modified BZT@MWCNTs/PB-1 composite film was reduced, and breakdown strength was improved. These changes were confirmed by the experimental results shown in Figure 8a,c.

### 3.6. Energy Storage Performance of PDA-Modified BZT@MWCNTs/PB-1 Composite Films

**Figure 10:**
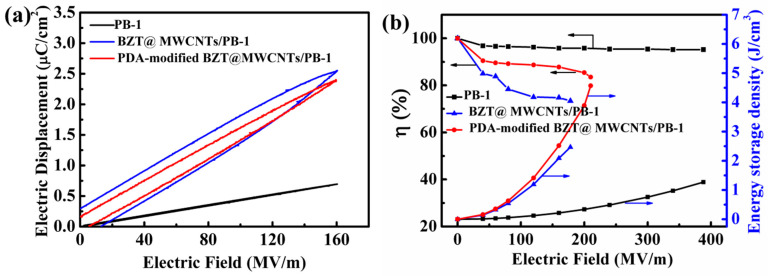
(**a**) Electric displacement electric field loops of the PDA-modified BZT@MWCNTs/PB-1 composite films under an applied field of 160 MV·m^−1^. (**b**) Energy storage density and energy storage efficiency at 1 kHz of the PDA-modified BZT@MWCNTs/PB-1 composite films.

The energy storage performance of PDA-modified BZT@MWCNTs/PB-1 composite films is shown in Figure 10. The electric displacement electric field loops (D–E loops) of the PDA-modified 10vol% BZT@0.5 vol% MWCNTs/PB-1 composite film were investigated and are illustrated in Figure 10a, wherein the D–E loops of pure PB-1 film and 10 vol% BZT@0.5 vol% MWCNTs/PB-1 composite film are provided for comparison. Under the applied field, the electric displacement of PB-1 followed a classic linear pattern and was increased by the addition of BZT@MWCNTs or PDA-modified BZT@MWCNTs nanoparticles. Under the same electric field, the maximum electric displacement of 10 vol% BZT@0.5 vol% MWCNTs/PB-1 and PDA-modified 10 vol% BZT@0.5 vol% MWCNTs/PB-1 composite films reached 2.54 and 2.38 μC/cm^2^, respectively, whereas that of PB-1 was as low as 0.69 μC/cm^2^. In addition, PDA modification could reduce remnant polarization due to the limited mobility of charge carriers. This decrement could be supported by a reduction in AC conductivity, as shown in Figure 8b.

Energy storage density and energy storage efficiency are key factors in the practical applications of dielectric capacitors. The energy storage density of all samples were calculated from D–E loops in accordance with the equation [42]:

U_e_ = ∫EdD. (7)

The formula for energy storage efficiency:

η = (discharge energy density/total energy density) × 100%.(8)

As shown in Figure 10b, at the electric field of 210 MV·m^−1^, the composite film with PDA-modified BZT@MWCNTs nanoparticles had higher energy storage density (4.57 J·cm^−3^) and discharge efficiency (83.5%) than the PB-1 film. Specifically, the energy storage density of this composite film was over 3.5 times that of PB-1 film (approximately 1.3 J·cm^−3^ at 388 MV·m^−1^). This result suggested that the PB-1-based composite film with PDA-modified BZT@MWCNTs could have potential applications in the design of pulse power capacitors with high energy density.

## 4. Conclusions

BZT@MWCNTs nanoparticles were synthesized by using a sol–gel hydrothermal method at 180 °C and then modified with PDA via noncovalent polymerization. A series of PB-1-based composite films were prepared via a solution-casting method by varying BZT@MWCNTs and PDA-modified BZT@MWCNTs contents. The dielectric constant of the composite films followed the order of BZT@MWCNTs/PB-1 > PDA-modified BZT@MWCNTs/PB-1 > MWCNTs/PB-1 > BZT/PB-1, and the dielectric loss of the composite films was in the order of MWCNTs/PB-1 > BZT@MWCNTs/PB-1 > PDA-modified BZT@MWCNTs/PB-1 > BZT/PB-1. This pattern suggested synergistic effects among BZT, MWCNTs, and PDA on the improved dielectric properties of PB-1 composite films. In addition, the PDA layer, which was used as the intermediate phase and insulating layer, could reduce dielectric loss and improve the breakdown strength of the PDA-modified BZT@MWCNTs/PB-1 composite film. The PB-1 composite film with PDA-modified 10 vol% BZT@0.5 vol% MWCNTs nanoparticles had the best dielectric performance. Its dielectric constant and dielectric loss were 25.43 and 0.0077 at 1 kHz, respectively, and its energy storage density was 4.57 J·cm^−3^. At the electric field of 210 MV·m^−1^, the energy storage efficiency of this film was 83.5%, which was over 3.5 times that of PB-1 film.

## Figures and Tables

**Table 1:** The dielectric performance of PB-1 composite films at 1 kHz.

Samples	ε’	tan δ (×10^−2^)	E_b_ (MV·m^−1^)	Ref.
PB-1	2.01	0.09	388	This work
10 vol% BZT/PB-1	4.40	0.36	212	This work
0.5 vol% MWCNTs/PB-1	4.92	7.01	138	[7]
10 vol% BZT@ 0.50 vol% MWCNTs/PB-1	28.40	1.40	178	This work
PDA-modified 10 vol% BZT@ 0.50 vol% MWCNTs/PB-1	25.43	0.77	210	This work

## Data Availability

The data presented in this study are available on request from the corresponding author.

## Supplementary Materials

The following are available online at https://www.mdpi.com/2073-4360/13/2/285/s1: Figure S1. The fracture surfaces morphology of the BZT/PB-1 composite film with different BZT content (a) 5 vol%, (b) 10 vol%, and (c) 15 vol%; Figure S2. (a) Heat flow (heating) and (b) heat flow (cooling) curves of the BZT/PB-1 composite films filled with different BZT content; Figure S3. Variation in (a) dielectric constant and (b) dielectric loss with frequency shown by the BZT/PB-1 composite films with different BZT contents. (c) Effects of the addition of different contents of BZT on the breakdown performance of the composite films; Table S1. Melting and crystallization parameters derived from the DSC measurements for the pure PB-1 and composite films with different BZT.
