# Supplementary material for: Significantly Improved Dielectric Performance of Poly(1-butene)-Based Composite Films via Filling Polydopamine Modified Ba(Zr0.2Ti0.8)O3-Coated Multiwalled Carbon Nanotubes Nanoparticles
Source: Polymers (Basel). 2021 Jan 17;13(2):285. doi: 10.3390/polym13020285 (PMC7830269; doi:10.3390/polym13020285)
Supplement: Supplementary file 1 [file polymers-13-00285-s001.pdf]

polymers

MDPI

Supplementary Materials

Significantly Improved Dielectric Performance of Poly(1-Butene)-Based Composite Films via Filling Polydopamine Modified $Ba(Zr_{0.2}Ti_{0.8})O_3$-Coated Multiwalled Carbon Nanotubes Nanoparticles

Lingfei Li [1], Qiu Sun [1,*], Xiangqun Chen [2], Zhaohua Jiang [1,*] and Yongjun Xu [1]

[1] School of Chemistry and Chemical Engineering, Harbin Institute of Technology, Harbin 150001, China; lilingfei3473@126.com (L.L.); xuyongjun1218@126.com (Y.X.)
[2] School of Materials Science and Engineering, Harbin Institute of Technology, Harbin 150001, China; chenxq@hit.edu.cn
* Correspondence: sunqiu@hit.edu.cn (Q.S.); jiangzhaohua@hit.edu.cn (Z.J.)

S1. The Fracture Surfaces Morphology of the BZT/PB-1 Composite Films

Fracture surfaces morphology of the composite film with different BZT content was characterized by SEM as shown in Figure S1. It could be observed in Figure S1a,b that the BZT nanoparticles had a good dispersion in the PB-1 matrix, as the amount of the BZT nanoparticles was no more than 10 vol% because of the BZT nanoparticles. When the amount of BZT was more than 15 vol%, as shown in Figure S1c, severe agglomeration of BZT nanoparticle were found in the PB-1 matrix, which could induce the deterioration of the dielectric properties of the composite film.

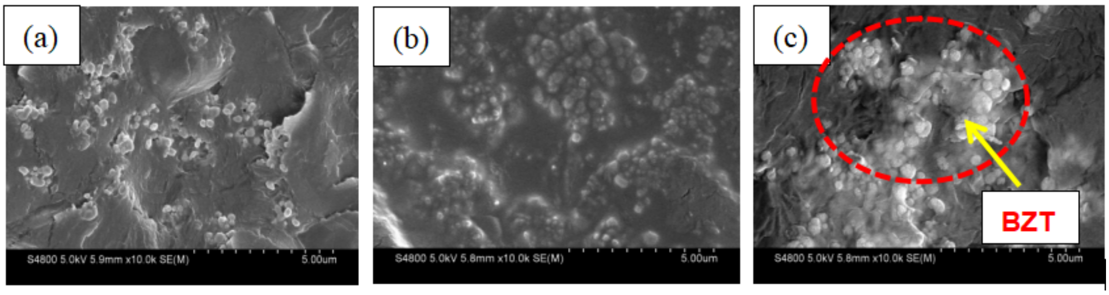

Figure S1. The fracture surfaces morphology of the BZT/PB-1 composite film with different BZT content (**a**) 5 vol%, (**b**) 10 vol%, and (**c**) 15 vol%.

S2. Heat Flow (Heating) and Heat Flow (Cooling) Curves of the BZT/PB-1 Composite Films

Compared with PB-1 film, the melting temperatures (T_m) of the composite films all elevated, the crystallization temperature (Tc) and Xc increased and then decreased with the increase of filler content BZT (Figure S2a,b and Table S1). The Xc of PB-1 film and BZT/PB-1 composite film are calculated using the following formula, and the results are listed in Table 1.

$$X_C = \Delta H_m \;/\; \omega\, \Delta H_m^0 \times 100\%, \tag{1}$$

where ΔH_m is the experimentally estimated melting enthalpy of pure PB-1 film and composite film, ΔH_m^0 is the melting enthalpy for 100% crystalline PB-1 (125.4 $J{\cdot}g^{-1}$), and ω is the weight fraction of PB-1. When the BZT content in the composite film was not more than 10 vol%, it indicated that the presence of BZT improved the crystallinity of the PB-1 film by heterogeneous nucleation. When the BZT content continued to increase to 15 vol%,

the Tc and Xc of the composite film decreased instead. A possible reason is that BZT agglomerates in the PB-1 matrix, resulting in a smaller space for the PB-1 molecular chain and weaker activity, which in turn leads to a decrease in Tc and Xc.

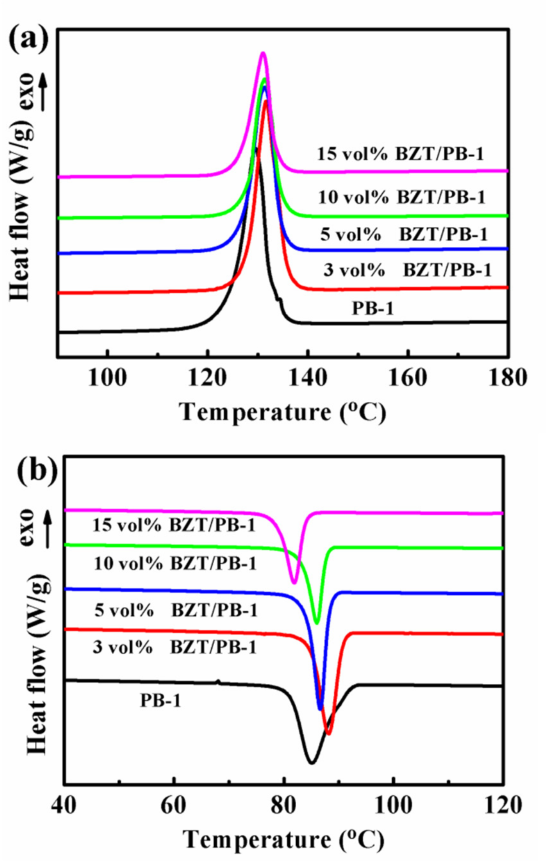

Figure S2. (**a**) Heat flow (heating) and (**b**) heat flow (cooling) curves of the BZT/PB-1 composite films filled with different BZT content.

Table S1. Melting and crystallization parameters derived from the DSC measurements for the pure PB-1 and composite films with different BZT.

Samples	Tm (°C)	ΔHm (J/g)	Tc (°C)	ΔHc (J/g)	Xc (%)
PB-1	129.54	71.09	85.11	40.39	56.7
3 vol% BZT/PB-1	131.69	69.22	88.18	33.68	59.4
5 vol% BZT/PB-1	131.38	65.24	86.65	30.53	57.8
10 vol% BZT/PB-1	131.36	57.69	86.00	20.64	57.5
15 vol% BZT/PB-1	131.05	38.98	81.91	20.65	44.4

S3. Dielectric Properties of BZT/PB-1 Composite Films

Frequency dependent behaviors of dielectric constant (ε') and dielectric loss (tan δ) of the PB-1 composite films with varied volume fraction of BZT were investigated, as shown in Figure S3. The ε' of all the BZT/PB-1 composite films remained basically unchanged between 10^2–10^5 Hz, which exhibited a similar trend with that of pure PB-1 film. When the volume fraction of BZT was 15 vol%, the ε' decreased slightly near the high frequency at 10^6 Hz. The reason might be that the rotation of the dipole was slower than the change of electric field, so the contribution of relaxation polarization, such as interfacial polarization, to the ε' was reduced slightly with the increase of frequency. In addition, the ε' of the PB-1 composite films increased with the increasing BZT nanoparticles, which could induce the interface polarization and then effectively enhance the local electric field in the polymer matrix. The ε' of the PB-1 composite film with 15 vol% BZT was highest at

100 Hz, i.e., 5.54, which was 2.8 times that of pure PB-1, as shown in Figure S3a. Compared with the pure PB-1 film, the tan δ of the composite film was almost unchanged below 10^4 Hz, except the sample with 15 vol% BZT, and it increased significantly with the increase of the BZT during 10^5–10^6 Hz. The high tan δ of the composite film with 15 vol% BZT might be caused by the accumulation of interface charges and greater interface loss induced by the agglomeration of BZT nanoparticles, as shown in Figure S3b.

The effects of BZT nano-fillers on the breakdown field strength of the PB-1 composite films were shown in Figure S3c. The tested β values of all samples were greater than 1, indicating that the test results are reliable. It was found that the breakdown field strength of the composite films decreased with the increasing BZT. It was greatly reduced to 128 $MV\cdot m^{-1}$ when the addition amount of BZT was 15 vol%, which was much lower than that of PB-1 (388 $MV\cdot m^{-1}$). The reason may be that though the adding fillers could give rise to defects in the polymer matrix, the low content of BZT filler had good dispersibility in the PB-1 polymer matrix, leading to only a little decrease of the breakdown field strength. When the amount of the BZT was 15 vol%, a large number of pores and deficiencies were found around the agglomerated BZT, and the shape factor β used to describe the uniformity and stability of the film was greatly reduced, leading to a sharp drop in breakdown strength. Thus, the sample with 10 vol% BZT was selected for the following work.

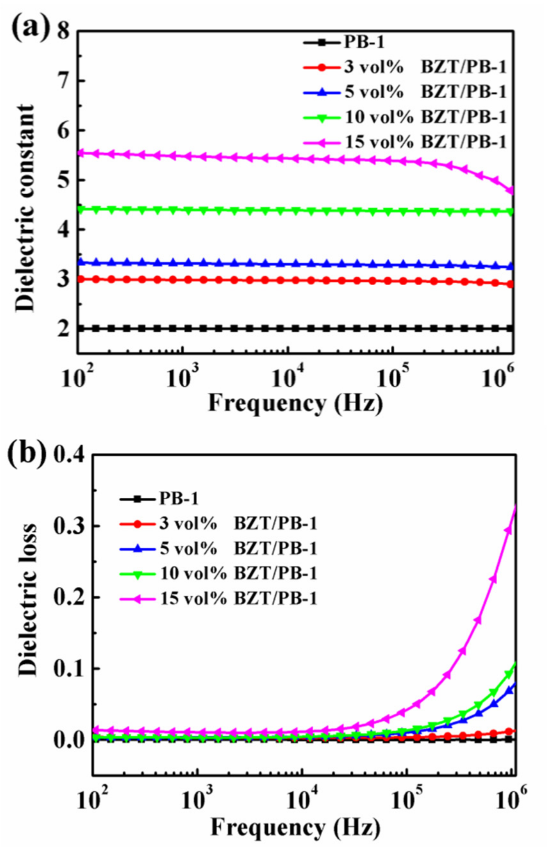

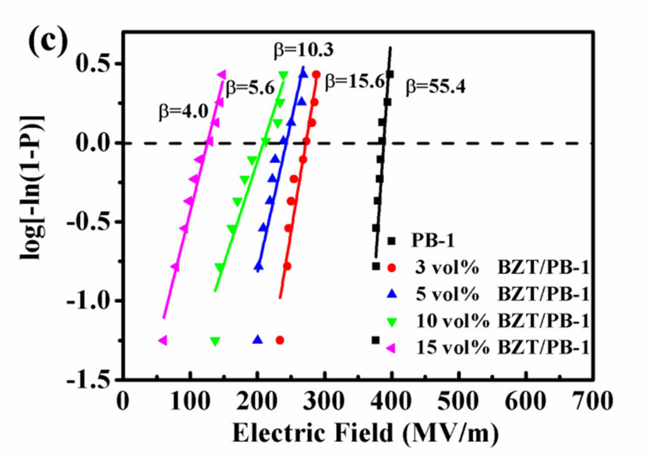

Figure S3. Variation in (**a**) dielectric constant and (**b**) dielectric loss with frequency shown by the BZT/PB-1 composite films with different BZT contents. (**c**) Effects of the addition of different contents of BZT on the breakdown performance of the composite films.
